# eIF2α Confers Cellular Tolerance to *S. aureus* α-Toxin

**DOI:** 10.3389/fimmu.2015.00383

**Published:** 2015-07-27

**Authors:** Gisela von Hoven, Claudia Neukirch, Martina Meyenburg, Sabine Füser, Maria Bidna Petrivna, Amable J. Rivas, Alexey Ryazanov, Randal J. Kaufman, Raffi V. Aroian, Matthias Husmann

**Affiliations:** ^1^University Medical Center, Institute of Medical Microbiology and Hygiene, Johannes Gutenberg-University, Mainz, Germany; ^2^Department of Pharmacology, Rutgers Robert Wood Johnson Medical School, Piscataway, NJ, USA; ^3^Degenerative Diseases Program, Sanford-Burnham Medical Research Institute, La Jolla, CA, USA; ^4^University of Massachusetts Medical School, Worcester, MA, USA

**Keywords:** pore forming toxins, *S. aureus* α-toxin, cellular tolerance, EIF2AK4, MAPK

## Abstract

We report on the role of conserved stress–response pathways for cellular tolerance to a pore forming toxin. First, we observed that small molecular weight inhibitors including of eIF2α-phosphatase, jun-*N*-terminal kinase (JNK), and PI3-kinase sensitized normal mouse embryonal fibroblasts (MEFs) to the small pore forming *S. aureus* α-toxin. Sensitization depended on expression of mADAM10, the murine ortholog of a proposed high-affinity receptor for α-toxin in human cells. Similarly, eIF2α*^S51A/S51A^* MEFs, which harbor an Ala knock-in mutation at the regulated Ser51 phosphorylation site of eukaryotic translation initiation factor 2α, were hyper-sensitive to α-toxin. Inhibition of translation with cycloheximide did not mimic the tolerogenic effect of eIF2α-phosphorylation. Notably, eIF2α-dependent tolerance of MEFs was toxin-selective, as wild-type MEFs and eIF2α*^S51A/S51A^* MEFs exhibited virtually equal sensitivity to *Vibrio cholerae* cytolysin. Binding of *S. aureus* α-toxin to eIF2α*^S51A/S51A^* MEFs and toxicity in these cells were enhanced as compared to wild-type cells. This led to the unexpected finding that the mutant cells carried more ADAM10. Because basal phosphorylation of eIF2α in MEFs required amino acid deprivation-activated eIF2α-kinase 4/GCN2, the data reveal that basal activity of this kinase mediates tolerance of MEFs to α-toxin. Further, they suggest that modulation of ADAM10 is involved. During infection, bacterial growth may cause nutrient shortage in tissues, which might activate this response. Tolerance to α-toxin was robust in macrophages and did not depend on GCN2. However, JNKs appeared to play a role, suggesting differential cell type and toxin selectivity of tolerogenic stress responses. Understanding their function or failure will be important to comprehend anti-bacterial immune responses.

## Introduction

Membrane perforation by pore forming toxins (PFT) is an ancient mode of attack employed by many bacteria, which helps them to establish or sustain infection (1–3). PFT represent a large group of bacterial toxins, which can be divided into various structural families (2). Many PFT have been discovered based on their ability not only to lyse red blood cells but they also affect nucleated cells, with effects ranging from induction of cell death to proliferation, time scales of occurrence from seconds to days after attack (4). Of particular relevance in the present context, cell autonomous defenses are in place to limit or reverse damage of nucleated target cells of PFT (4–13); they have been discussed as an integral part of the innate immune system, defending against bacteria (14). Work in *C. elegans* identified MAPK as master regulators of defense against PFT (7, 15). Whereas the importance of p38 MAPK is well established (3, 4, 9, 16–18), data on the role of jun-*N*-terminal kinases (JNKs) are somewhat conflicting (19–21). Large scale analyses of perforated cells have identified multiple additional changes taking place in response to PFT (15, 20, 22, 23), many of which appear to be triggered by the drop of cytosolic potassium (11, 20, 24, 25). Although the contribution of the various pathways to cell autonomous defense against PFT remains to be established in most cases, a basic concept emerges according to which removal of membrane pores (3, 10, 13, 26–32) and metabolic homeostasis (10, 12, 13, 20, 33, 34) are cornerstones of early cell autonomous defense against PFT. Importantly, mechanisms involved in pore removal depend on PFT and cell type (4, 8, 29). It appears that MAPK p38 and autophagy are required if the recovery process is prolonged as with *S. aureus* α-toxin and aerolysin (8, 20).

Phosphorylation of eukaryotic translation initiation factor 2 α (eIF2α) is a conserved stress–response activated by various PFT (12, 13, 20, 33–35). How this pathway impacts survival of target cells remains incompletely understood. Eukaryotic translation initiation requires assembly of a 43S ternary pre-initiation complex, consisting of met-t-RNAi(Met), eIF2, and GTP. In mammalian cells, this step is controlled through phosphorylation of eIF2α at serine 51 by 1 of 4 eIF2α-kinases (GCN2, PERK, PKR, and HRI), which respond to different types of stress (36). GCN2 serves as nutritional sensor, which is activated by uncharged t-RNAs (37, 38). Several lines of evidence indicate that membrane stress triggers this pathway: first, mutations that affect vesicular transport in yeast trigger phosphorylation of eIF2α (39). Second, in mammalian cells, plasma membrane perforation by bacterial PFT leads to activation of GCN2 (12, 33), phosphorylation of eIF2α, transient attenuation of translation, and activation of autophagy (12, 13, 20). Also, membrane damage by chlorpromazine or detergent triggers GCN2 (40). *S. aureus* α-toxin inhibited uptake of leucine by cells, providing an explanation for activation of GCN2 in target cells of PFT (12). In human epithelial cells, eIF2α, eIF2α-kinases and the regulatory eIF2α-phosphatase subunit CReP/Ppp1r15B are all required for efficient recovery from α-toxin-dependent loss of ATP (13). Surprisingly, these proteins served to remove membrane pores, thus, linking control of translation initiation and membrane traffic (13).

Conspicuously, many rodent cell types are not affected even by comparably high concentrations (micromolar range) of α-toxin; but the cause is not known. Receptor density on murine cells might be low, or murine ADAM10 might be an inefficient receptor as compared to its human counterpart. Alternatively, murine cells might be particularly tolerant to the consequences of successful attack. At any rate, to better understand results of *in vivo* experiments with *S. aureus* or α-toxin in mice, it is important to comprehend the mechanisms underlying tolerance of murine cells.

In ecoimmunology, “tolerance” denotes the ability of an organism to cope with high-pathogen load and resulting damage (41–43). To elucidate mechanisms of tolerance, it will be important to investigate the phenomenon at the cellular level using defined noxious agents. Here, we focus on cellular tolerance to PFT. A PFT may fail to cause overt damage to a cell if it cannot bind to, or attack, membranes in the first place. Alternatively, target cells may be able to cope with membrane perforation. In both cases, we consider the target cell “tolerant to the PFT.” The term “susceptibility” shall denote responsiveness of a cell to a PFT as measured by loss of ATP or loss of potassium ions from the cytosol.

In the present work, we have investigated tolerance of mouse cells to *S. aureus* α-toxin. The results support a broader protective function of MAPK and document a cell type- and toxin-selective role of eIF2α.

## Results

### Normal mouse embryonal fibroblasts are tolerant to *S. aureus* α-toxin

**Figure 1:**
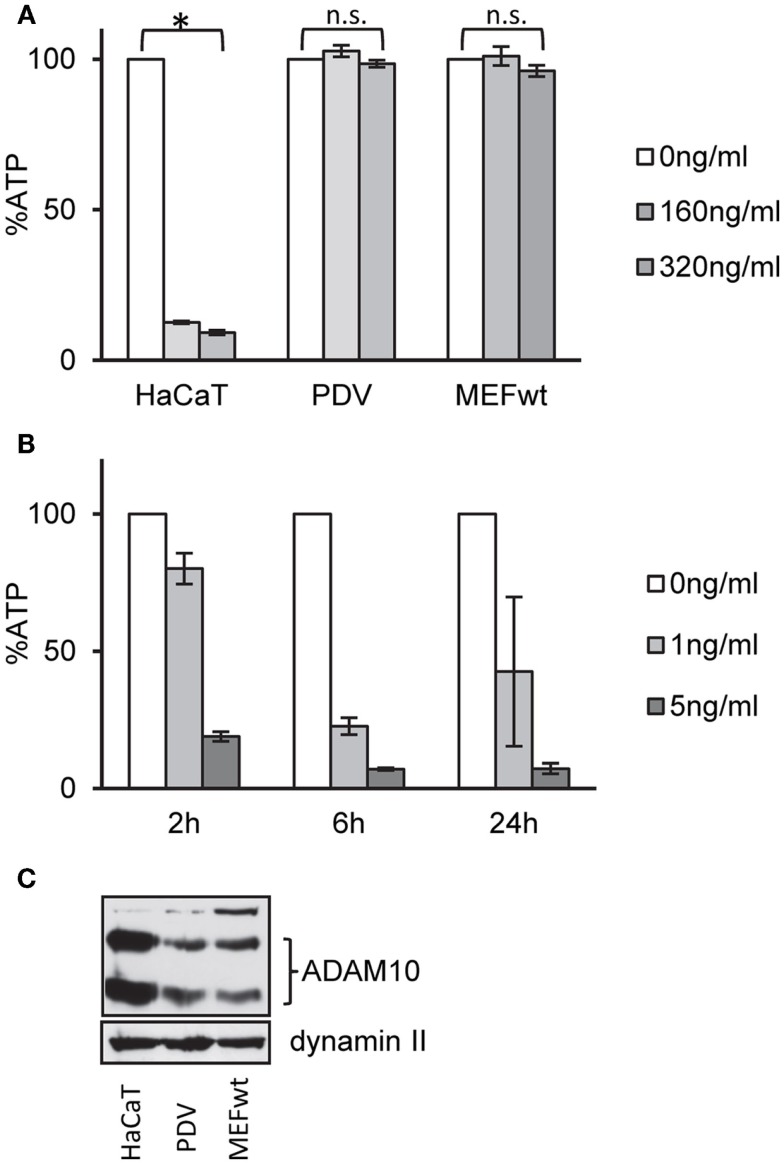
**Wild-type MEFs are tolerant to α-toxin**. **(A)** HaCaT cells (human keratinocytes), PDV cells (murine keratinocytes), or MEFs wt cells (mouse embryonal fibroblasts) were treated or not with α-toxin. Cellular ATP levels were determined after 2 h; data are percent of untreated controls; mean values ±SE; *n* ≥ 4; asterisk denotes *p* ≤ 0.05. **(B)** MEFs wt cells were treated with indicated doses of pVCC and cellular ATP levels were determined after 2, 6, or 24 h; shown are percent of untreated controls; mean values ±SE; *n* = 3. **(C)** Cell lysates of wt, PDV, and HaCaT were analyzed by Western blot for ADAM10 and dynamin II (loading control).

Exposure of human keratinocytes (HaCaT) to nanomolar concentrations of α-toxin for 2 h leads to significant loss of ATP. In contrast, murine keratinocytes (PDV) or mouse embryonal fibroblasts (MEFs) appeared to be not susceptible to α-toxin (Figure 1A). In contrast, MEFs were exquisitely susceptible to pVCC (Figure 1B), another PFT of the small β-barrel family (44).

### Inhibitors of various signaling pathways sensitize MEFs for α-toxin

**Figure 2:**
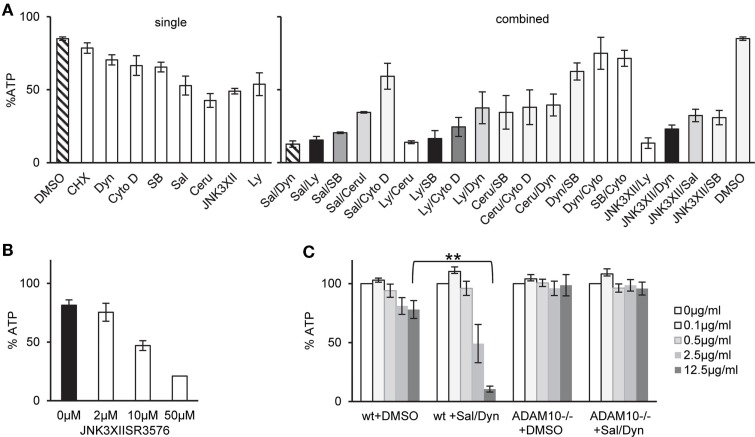
**Inhibitors of various pathways sensitize MEFs to α-toxin**. **(A)** wt cells were treated as indicated either with single inhibitors or combinations of two inhibitors listed below and treated with 10 μg/ml α-toxin. Cellular ATP levels were determined after 2 h; data are percent of untreated controls; shown are mean values for single inhibitors ±SE with *n* ≥ 6, or mean values of *n* ≥ 2 for combinations of inhibitors, SE was <30% throughout. Inhibitor concentrations: Salubrinal (40 μM), Dynasore (80 μM), Ly29400L (100 μM), SB203580 (20 μM), Cerulenin (20 μM), Cytochalasin D (20 μM), and JNK3XIISR3576 (10 μM). **(B)** MEFs wt were treated with indicated doses of JNK3XIISR3576 and incubated with 10 μg/ml α-toxin. Cellular ATP levels were determined after 2 h; data are percent of controls treated with similar concentrations of JNK3XIISR3576 but not treated with α-toxin; mean values ±SE; *n* ≥ 3. **(C)** MEFs wt or MEFs ADAM10^−/−^ (mouse embryonal fibroblasts) were pretreated with 40 μM Salubrinal and 80 μM Dynasore or solvent alone for 30 min and treated or not with indicated doses of α-toxin. ATP levels were determined after 2 h; shown are percent of untreated controls; mean values ±SE; *n* = 3; two asterisks denote *p*-values ≤0.001.

One potential explanation for selective tolerance of MEF to α-toxin could be the lack of an appropriate receptor, or lower expression levels of receptor. Amino acid sequences of human and murine ADAM10, the proposed high-affinity receptor of α-toxin, are not identical. Western blots with an antibody against ADAM10 yielded weaker bands with the murine cells (Figure 1C). Although it cannot be excluded that the antibody binds human and murine cells with different efficiency, it is therefore possible that qualitative or quantitative differences in receptor expression could explain lower susceptibility of murine vs. human cells to α-toxin. Alternatively, however, efficient ongoing repair of membrane damage and fitness to cope with metabolic stress could also play a role. Therefore, we tested a panel of small molecular weight compounds that we knew to inhibit recovery from α-toxin-dependent ATP-depletion in HaCaT cells. Several (combinations of) inhibitors sensitized MEFs for α-toxin (Figure 2A). The effect depended on the concentration of inhibitor, as exemplified for JNK3XIISR3576 (Figure 2B). Although JNK3XIISR3576 is selective for JNK3 if applied at nanomolar concentrations, MAPK other than JNK3 seem to be involved here because MEFs do not express JNK3 (45). As shown for Sal/Dyn, inhibitors sensitized wild-type MEF for α-toxin, provided they expressed ADAM10 (Figure 2C). Thus, inhibitors did not sensitize cells by increasing unspecific toxicity of α-toxin.

### Lack of phosphorylatable eIF2α, GCN2, or Ppp1r15B sensitizes MEFs for α-toxin

That Salubrinal, an inhibitor of eIF2α-phosphatases (46) sensitized MEFs to α-toxin, and similar observations in keratinocytes (13), prompted us to investigate the response of MEFs with defined genetic modifications affecting expression or function of proteins involved in regulation of translation: first, in eIF2α*^S51A/S51A^* cells, the eIF2α locus is replaced with a non-phosphorylatable version, thus precluding regulated attenuation of translation via P-eIF2α. Second, GCN2^−/−^ MEFs lack nutrient sensitive eIF2α-kinase GCN2/EIF2AK4, thereby blunting phosphorylation of eIF2α in response to amino acid deprivation. Third, Ppp1r15B^−/−^ MEFs are devoid of the sole constitutive regulatory subunits of eIF2α-phosphatase (47); this defect leads to constitutively higher eIF2α-phosphorylation levels. Fourth, EeF2K^−/−^ MEFs lack eukaryotic elongation factor 2 kinase (EeF2K), which functions downstream of mTOR to control protein synthesis (48).

**Figure 3:**
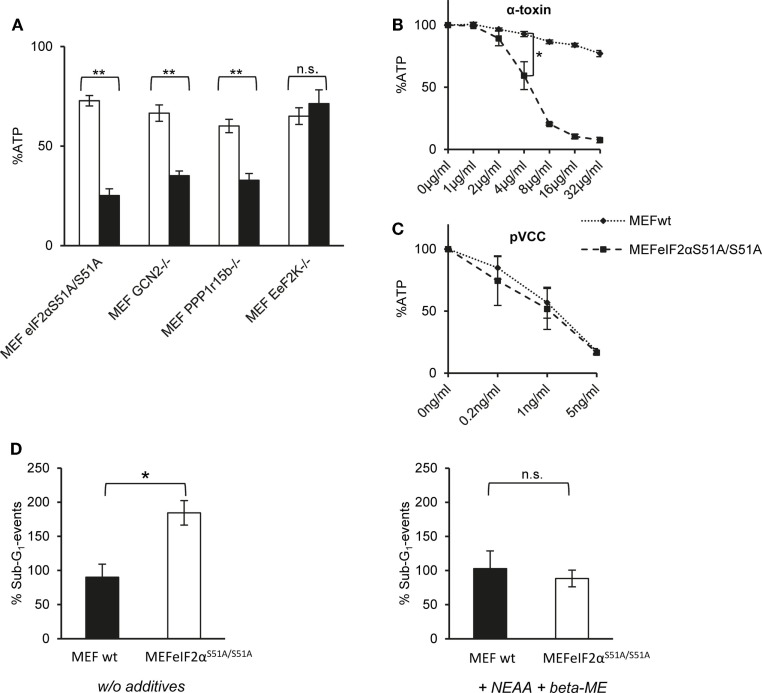
**Dysregulation of eIF2α-phosphorylation increases sensitivity for α-toxin**. **(A)** MEFs eIF2α^S51A/S51A^, MEFs GCN2^−/−^, MEFs Ppp1r15b^−/−^, or MEFs EeF2K^−/−^ were treated or not with 10 μg/ml α-toxin. ATP levels were determined after 2 h; shown are percent of untreated controls; mean values ±SE; *n* ≥ 4. Black bars show data with MEFs cell variants as indicated; white bars corresponding control cells; two asterisks denote *p*-values ≤0.001. **(B)** MEFs wt or eIF2α^S51A/S51A^ were treated, or not with indicated doses of α-toxin. Cellular ATP levels were determined after 2 h; data are percent of untreated control; mean values ±SE; *n* = 5; asterisk: *p* = 0.026. **(C)** MEFs wt or MEFs eIF2α^S51A/S51A^ treated or not with indicated doses of pVCC. Cellular ATP levels were determined after 2 h; data are percent of untreated control; mean values ±SE; *n* = 3. **(D)** MEFs wt or MEFs eIF2α^S51A/S51A^ were cultured in standard media or in presence of β-ME and additional non-essential amino acids and treated with 10 μg/ml α-toxin for 48 h, stained with Propidium iodide and subsequently frequency of Sub-G_1_-DNA was determined; data show percent of untreated controls; mean values ±SE; *n* = 3; asterisk denotes *p* ≤ 0,05.

First, we compared ATP levels in these MEFs to assess metabolic perturbation after treatment with α-toxin. EeF2K^−/−^ cells were not susceptible to 10 μg/ml of α-toxin and thus behaved like wild-type MEFs. In contrast, lack of GCN2 or Ppp1r15B both sensitized cells to α-toxin. The strongest effect was observed with eIF2α*^S51A/S51A^* MEFs, which lost up to ~80% of ATP (Figure 3A). Although α-toxin led to dose-dependent loss of ATP in eIF2α^S51A/S51A^ cells, there was barely any effect on wild-type cells (Figure 3B). Notably, pVCC-dependent loss of ATP was similar in wild-type or mutant MEFs (Figure 3C). Next, we determined the frequency of sub-G1 events, a measure of DNA fragmentation, in cells treated with α-toxin for 48 h. No significant difference was observed between wild-type and eIF2α*^S51A/S51A^* cells in the presence of β-ME and non-essential amino acids (NEAA). However, without these supplements, the number of sub-G1 events was doubled in eIF2α*^S51A/S51A^* cells (Figure 3D), although toxin-dependent loss of ATP and phosphorylation of eIF2α were equal in media with or without additives (data not shown). Additives thus appear to protect cells from secondary damage.

### GCN2/EIF2AK4 contains α-toxin-dependent stress

**Figure 4:**
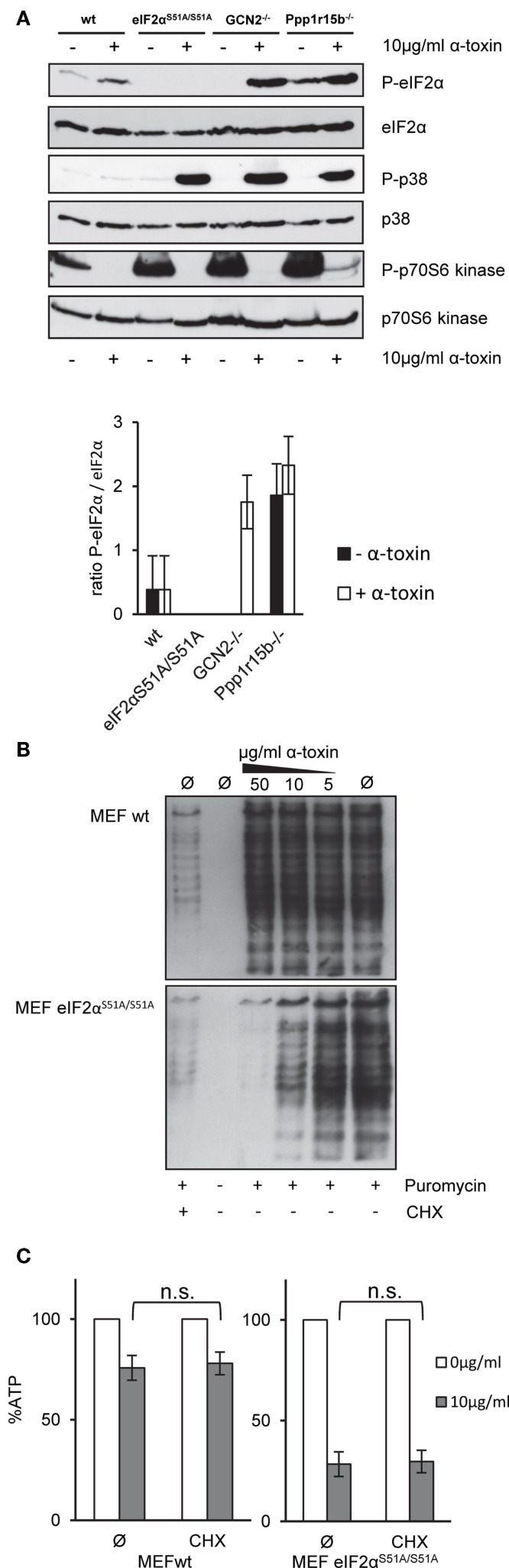
**α-toxin causes translational arrest in MEFs eIF2α^S51A/S51A^**. Cycloheximide does not sensitize wild-type MEFs **(A)** MEFs wt, MEFs eIF2α^S51A/S51A^, MEFs GCN2^−/−^, or MEFs Ppp1r15b^−/−^ were treated or not with 10 μg/ml α-toxin for 2 h. Cell lysates were analyzed by Western blot for P-eIF2α, eIF2α, P-p70S6 kinase, p70S6 kinase P-p38, and p-38. One of three similar blots is shown; lower panel summarizes densitometric data for (P)-eIF2α, mean ± SE; *n* = 3 **(B)** MEFs wt or MEFs eIF2α^S51A/S51A^ cells were treated or not with indicated concentrations of α-toxin and incubated for 1 h at 37°C. Treatment with CHX served as positive control for translational arrest. Subsequently, the cells were incubated for 1 h at 37°C with 10 μg/ml puromycin, which incorporates during ongoing synthesis into nascent proteins. Eventually, cells were analyzed by Western blot for puromycin. **(C)** MEFs wt or MEFs eIF2α^S51A/S51A^ cells were treated or not with 10 μg/ml α-toxin and incubated with or without CHX at 37°C. Cellular ATP levels were determined after 2 h; data are percent of untreated controls; mean values ±SE; *n* = 5.

Next, we assessed basal or toxin-dependent eIF2α-phosphorylation in the various MEFs lines. In wild-type cells, some basal phosphorylation of eIF2α was noted, which was moderately increased by α-toxin; after normalization for eIF2α, the effect was, however, statistically insignificant. As expected, P-eIF2α was not detected in eIF2α*^S51A/S51A^* MEFs (Figure 4A). No P-eIF2α was also discerned with samples of untreated GCN2^−/−^ cells; this indicated that basal phosphorylation of eIF2α in cultured MEFs depends on GCN2. Paradoxically, however, α-toxin-dependent phosphorylation of eIF2α was enhanced in cells lacking this eIF2α-kinase. This showed that increased susceptibility of GCN2^−/−^ MEFs to α-toxin (Figure 3A) cannot be accounted for by diminished toxin-dependent eIF2α-phosphorylation. Membranes were re-probed with antibodies against p38, which becomes phosphorylated in response to PFT. Strikingly, α-toxin caused robust phosphorylation of stress activated protein kinase p38 in GCN2^−/−^ MEFs, Ppp1r15B MEFs, and eIF2α*^S51A/S51A^* MEFs (Figure 4A), but not in wild-type MEFs, supporting the notion that cells with imbalanced eIF2α-phosphorylation experienced more severe toxin-dependent stress. p70S6K, substrate of mTORC1 became de-phosphorylated (Figure 4A), indicating that α-toxin inhibits mTORC1, master regulator of translation and autophagy.

### CHX does not tolerize eIF2α^*S51A/S51A*^ MEFs to α-toxin

Results from Western blots (Figure 4A) raised the question whether α-toxin impacts translation differentially in eIF2α*^S51A/S51A^* vs. wild-type MEFs. Paradoxically, treatment with α-toxin caused dose-dependent attenuation of translation in eIF2α*^S51A/S51A^* MEFs, but had no such effect on wild-type MEFs (Figure 4B). This suggested that translation was inhibited in response to α-toxin through a mechanism that was independent of eIF2α-phosphorylation. Together with results from the foregoing ATP-assays, this also revealed that attenuation of translation *per se* is insufficient to maintain metabolic homeostasis upon attack by α-toxin. Conversely, toxin-dependent ATP-loss in eIF2α*^S51A/S51A^* MEFs is not a consequence of translational arrest, because treatment of wild-type cells with CHX stops translation (Figure 4B), but does not hyper-sensitize MEFs for α-toxin (Figure 4C).

### Wild-type eIF2α modulates binding and action of α-toxin

**Figure 5:**
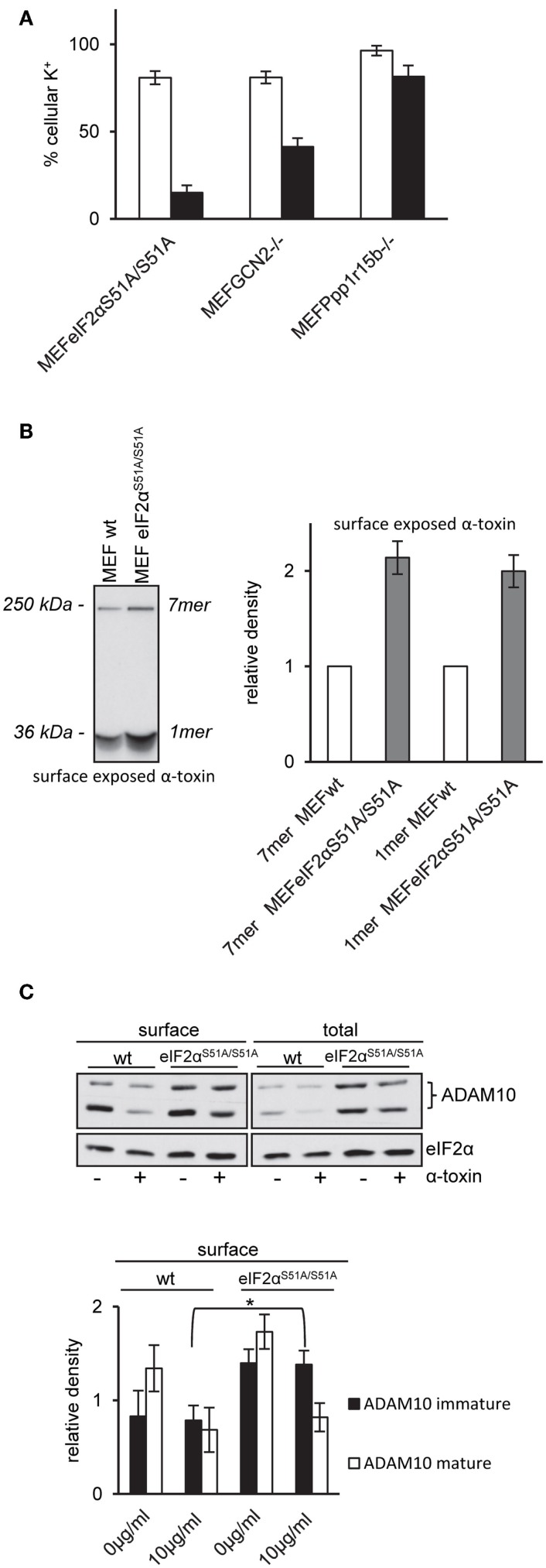
**MEFs eIF2α^S51A/S51A^ over-express ADAM10 and bind higher amounts of α-toxin**. **(A)** MEFs eIF2α^S51A/S51A^, MEFs GCN2^−/−^, MEFs Ppp1r15b^−/−^, or corresponding control cells were treated or not with 10 μg/ml α-toxin. Potassium levels were determined after 2 h, shown are percent of untreated controls; mean values ±SE; *n* ≥ 4. Black bars show data of MEFs cell variants as indicated; white bars corresponding control cells. **(B)** Right: MEFs wt or MEFs eIF2α^S51A/S51A^ cells were incubated with radio-labeled α-toxin 2 and 8 μg/ml α-toxin at 37°C for 15 min. Subsequently, cells were surface biotinylated, lysates were obtained and subjected to sequential neutravidin-pulldown (NP) and immunoprecipitation (IP) followed by PAGE/fluography, as described in Kloft et al. (13). Left: band intensities were measured by densitometry using ImageJ software. Shown are mean values ±SE; *n* = 4. Variations of loading with toxin and plating of cells were <1 and <10%, respectively. **(C)** MEFs wt or MEFs eIF2α^S51A/S51A^ were treated or not with 10 μg/ml α-toxin for 2 h. Subsequently, cells were surface biotinylated or not and lysed. Lysates were subjected to NP; both lysates and precipitation were analyzed by Western blot for ADAM10 and eIF2α (loading control); upper panel: one of four similar blots; lower panel: bar chart summarizing data (mean ± SE; *n* = 4).

That inhibition of translation did not protect eIF2α*^S51A/S51A^* MEFs provoked the question how phosphorylation of eIF2α tolerizes MEF. So, we investigated a primary event underlying many of the rapid molecular changes induced by PFT, i.e., disturbance of natural ion gradients (4). Loss of potassium appears to be one major trigger (11, 20, 24, 49). We measured loss of intracellular potassium by flame photometry. In line with results from ATP-assays, net loss of potassium was enhanced in GCN2^−/−^ or Ppp1r15B^−/−^ cells and even more so in eIF2α*^S51A/S51A^* MEFs (Figure 5A), suggesting that membrane damage was more severe in cells with deficiencies in regulation of eIF2α-phosphorylation.

Pore formation by α-toxin depends on oligomerization and insertion into the plasma membrane. Because oligomers resist SDS, they can be detected by SDS-PAGE. Using cell surface labeling after incubation with internally radio-labeled α-toxin, we compared the amount of α-toxin on the surface of wild-type MEFs and eIF2α*^S51A/S51A^* MEFs. The fluorographic analysis shown in Figure 5B reveals that more α-toxin is present at the surface of eIF2α*^S51A/S51A^* MEFs as compared to wild-type cells, providing a straightforward explanation for enhanced loss of potassium and ATP from these cells.

### Wild-type eIF2α modulates expression of ADAM10

Increased amounts of toxin associated with eIF2α*^S51A/S51A^* MEFs could be due to higher abundance of α-toxin receptors. Therefore, we compared ADAM10 expression in eIF2α*^S51A/S51A^* MEFs and wild-type MEFs. Western-blot analysis revealed that ADAM10 is over-expressed in eIF2α*^S51A/S51A^* MEFs; and more ADAM10 is exposed on the cell surface of eIF2α*^S51A/S51A^* MEFs as compared to wild-type cells (Figure 5C). No significant differences between wild-type cells and eIF2α*^S51A/S51A^* MEFs were found in a lipidomics analysis (data not shown). Treatment with α-toxin led to down-regulation of ADAM10 at the cell surface of both wild-type and mutant cells. Because basal eIF2α-phosphorylation in MEFs depends on GCN2 (Figure 4A), the collective data indicate that nutrient stress or basal activity of GCN2 modulates levels of ADAM10 in MEFs.

### Role of GCN2 for tolerance to α-toxin is not conserved in BMDM

**Figure 6:**
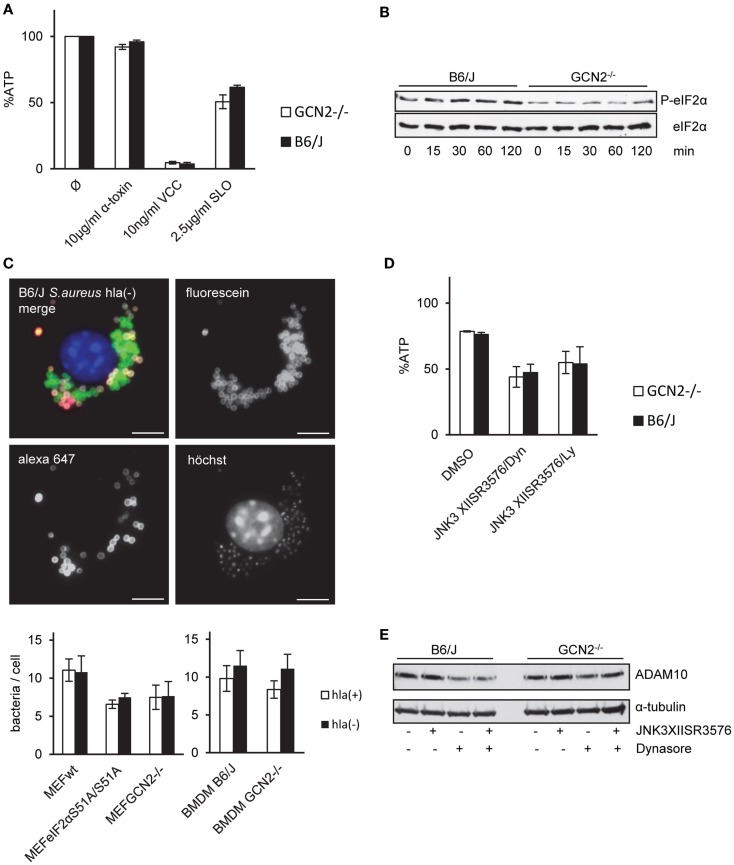
**GCN2 does not render BMDMs tolerant to α-toxin**. **(A)** BMDMs isolated from GCN2^−/−^ mice or control mice (B6/J) were treated or not with indicated doses of α-toxin, VCC, or SLO. Cellular ATP levels were determined after 2 h. White bars show BMDMs from GCN2^−/−^ mice and black bars show BMDMs from control mice; mean values ±SE, *n* ≥ 3 **(B)** BMDMs of GCN2^−/−^ mice or control mice were incubated with 10 μg/ml α-toxin for indicated times. Cell lysates were analyzed by Western blot for P-eIF2α and eIF2α. **(C)** MEFs variants (right graph) or BMDMs of GCN2^−/−^ and control mice (left graph) were incubated with fluorescein/biotin labeled *S. aureus* hla(−) or hla(+) strains (MOI 1:30) for 1 h, washed and incubated for an additional hour at 37°C. After fixation, extracellular bacteria were stained with streptavidin-coupled Alexa 647. Upper: exemplary picture of solely fluorescein-stained *S. aureus*, representing intracellular bacteria, and extracellular *S. aureus* that were accessible for Alexa 647-Streptavidin. Lower: graphs show counts of solely fluorescein-stained (intracellular) bacteria per cell; mean values ±SE; BMDMs *n* = 2; MEFs *n* = 3. **(D)** BMDMs of GCN2^−/−^ mice or control mice were incubated with combinations of JNK3XIISR3576 (10 μM), Dynasore (80 μM), JNK3 XIISR3576 (10 μM), and Ly29400L (100 μM) or solvent alone and treated with 10 μg/ml α-toxin. Cellular ATP levels were determined after 2 h (percentage of controls). (Mean values ±SE; *n* = 3). **(E)** BMDMs of GCN2^−/−^ and control mice were treated with the combination of Dynasore (80 μM) and JNK3XIISR3576 (10 μM), Dynasore (80 μM) alone, JNK3XIISR3576 (10 μM) alone, or solvent alone (DMSO) for 2.5 h. Cell lysates were analyzed by Western blot for ADAM10 and α-tubulin (loading control).

Like MEFs, BMDM proved to be highly tolerant to α-toxin although they were susceptible to *Vibrio cholera* cytolysin (VCC) (Figure 6A). However, lack of GCN2-expression in BMDM did not significantly alter susceptibility to α-toxin (Figures 6A,D); α-toxin-dependent phosphorylation of eIF2α appeared slightly reduced (Figure 6B). Further, infection of BMDM by *S. aureus* was equally efficient with both strains whether or not bacteria produced toxin or not (Figure 6C). Therefore, GCN2 seemed to play no major role for resistance or tolerance to α-toxin of BMDMs.

Small MW inhibitors of JNK and dynamin, a cocktail, which efficiently breaks tolerance to α-toxin in MEFs, moderately sensitized BMDM to purified α-toxin (Figure 6D). Inhibitors did not enhance ADAM10 expression (Figure 6E). Thus, MAPK enhance natural tolerance of BMDM to α-toxin, but modulation of ADAM10 expression appears not to be involved.

## Discussion

One conclusion of this work is that regulators of translation initiation (eIF2α, GCN2, and Ppp1r15B) render murine embryonic fibroblasts tolerant to *S. aureus* α-toxin. This is consistent with our previous finding in human epithelial cells that these proteins promote recovery from successful attack (13). Although it remains to be investigated whether *a priori* tolerance of MEFs to α-toxin is likewise based on efficient endocytic removal of α-toxin pores from the cell surface, the present results document that the tolerogenic effect of eIF2α, GCN2, and Ppp1r15B is conserved in mice and man, and that it is observable in fibroblasts. That both lack of an eIF2α-kinase and of constitutive eIF2α-phosphatase reduce tolerance to α-toxin could be interpreted to show that balanced phosphorylation of eIF2α, or cycling of eIF2α between phosphorylated and unphosphorylated state is required for the tolerogenic effect.

A role of translational regulation for various aspects of innate immunity, and a protective function against PFT has been discussed in the recent literature (34, 50, 51). One established function of eIF2α-phosphorylation and the integrated stress–response is to reprogram expression of genes, including of genes that regulate immunity. Translational attenuation in host cells might also help them to conserve energy, as proposed by some authors (20). However, (P)-eIF2α-dependent tolerance of MEF could not be explained by toxin-dependent translational attenuation *per se*: actually, α-toxin caused inhibition of translation in eIF2α*^S51A/S51A^* MEFs, but not in wild-type MEFs, and CHX did neither protect nor hyper-sensitize wild-type cells from/for α-toxin. Yet, eIF2α*^S51A/S51A^* MEFs proved to be significantly less tolerant to α-toxin. Probably as a consequence, protein synthesis was halted through alternative pathways. This could occur, for instance, by deactivation of mTOR, as indeed suggested by α-toxin-dependent dephosphorylation of p70S6K, a target of mTOR. The data may reflect a hierarchy of stress responses: Activation of eIF2α via nutritional sensor GCN2 contains α-toxin-dependent damage and stress, which would otherwise lead to exaggerated hyper-phosphorylation of eIF2α by (an)other eIF2α-kinase(s), possibly PERK and PKR. If eIF2α-phosphorylation fails, translation would be halted via robust deactivation of mTOR. Sustained inhibition of translation is obviously not tolerated by cells (35), but marked α-toxin-dependent inhibition of translation, observed in eIF2α*^S51A/S51A^* MEFs, does not explain the increased loss of ATP and potassium from these cells, because CHX did not affect these parameters in wild-type cells.

The fact that lack of phosphorylatable eIF2α was associated with increased toxin-dependent loss of potassium and ATP prompted us to measure the binding of α-toxin and expression of ADAM10, the proposed high-affinity receptor for α-toxin (52). This led to the unexpected finding that ADAM10 levels were significantly higher in eIF2α*^S51A/S51A^* cells. Therefore, tolerance to α-toxin in MEFs may be well due to GCN2/P-eIF2α-dependent modulation of its receptor, ADAM10. Whether or not the apparent link between basal nutrient stress and expression of ADAM10 was shaped by co-evolution of *S. aureus* and humans, it may also bear on functions of ADAM10 that are not related to infection.

**Figure 7:**
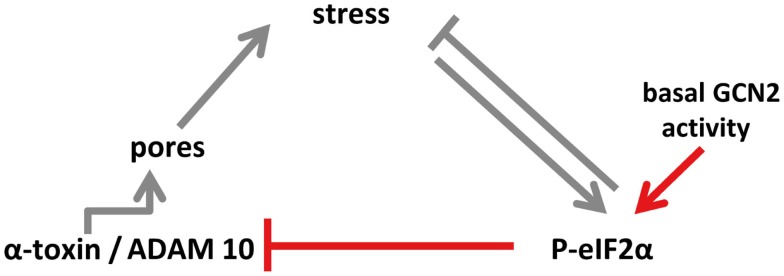
**Model of eIF2α-dependent cellular tolerance to α-toxin**. Disruption of membrane integrity by insertion of membrane pores causes various forms of stresses in target cells (e.g., loss of potassium, starvation), which trigger an array of conserved responses, including phosphorylation of MAPK and of eIF2α. Not only may these pathways feed back to alleviate stress but also eIF2α may modulate formation of *S. aureus* α-toxin membrane pores (this work; highlighted in red in the scheme), or persistence of lesions (13), root causes of α-toxin-dependent stress. Notably, basal activity of GCN2 maintains low levels of ADAM10, resulting in bated binding of α-toxin. Thus, basal nutrient stress in cells could serve as a preemptive stimulus of cellular tolerance to *S. aureus* α-toxin. This link, which might have evolved from mutual adaptation of *S. aureus* and humans, is selective, as suggested by the fact that *Vibrio cholerae* cytolysin breaks cell autonomous defense.

How basal phosphorylation of eIF2α-levels modulates ADAM10-expression remains to be elucidated. Because P-eIF2α is required for starvation-dependent autophagy (53), a possible role of eIF2α in this context could be to maintain basal autophagic flux, which in turn could impact ADAM10 levels. Recently, Atg16L1, a protein essential for autophagy, has been shown to confer tolerance to α-toxin; the authors proposed that autophagy constitutively dampens ADAM10 levels in a cell-type selective manner (54). Similarly, eIF2α-dependent modulation of ADAM10 shown in the present work is constitutive and cell-type selective. Together this seems to suggest that basal eIF2α-phosphorylation functions upstream of autophagy to mediate tolerance. Alternatively, P-eIF2α, eIF2α-kinases, and -phosphatases could function through mechanisms acting in parallel to autophagy, for instance, by regulating endocytosis of membrane pores, as has been shown in epithelial cells (13). Whatever the effector mechanism(s) downstream of eIF2α are, our data reveal that GCN2-dependent basal phophorylation of eIF2α in MEF modulates ADAM10 levels as well as binding and action of α-toxin. Because GCN2 is activated by low levels of amino acids in cells, basal nutrient stress might be the driving force; the potential links discussed here are summarized in a model (Figure 7).

Inhibitors of various pathways hyper-sensitized MEFs to *S. aureus* α-toxin, which supports the notion that multiple signaling pathways are required to confer cellular tolerance to α-toxin. This raises the possibility that drugs used in pharmaco-therapy may have potential tolerance-modulating effects, an issue that warrants further investigation. The present data support a tolerogenic role of JNKs in both MEFs and BMDM. More work is required to understand the underlying mechanisms, but they seem to be distinct from eIF2α-dependent cellular tolerance to α-toxin. Conserved stress responses may fail to protect against some PFT, as exemplified here by *V. cholerae* cytolysin. Whether cells of the immune system tolerate attack of a given PFT will co-determine the ability of an organism to mount an effective “classic” immune response to infection with corresponding bacteria.

## Materials and Methods

### Antibodies

Antibodies against P-eIF2α (phosphorylated at Ser51), eIF2α, P-p38 (phosphorylated at Thr180/Tyr182), p-38, P-p70S6K (phosphorylated at Thr389), p70S6K, ADAM10, and α-tubulin were from Cell Signaling Technology. Antibodies against dynamin II were purchased from Santa-Cruz Biotechnology. Anti-Puromycin-antibody was from Merck Millipore. Antibodies against LC3 were bought from Sigma. HRP-conjugated secondary antibodies were from Santa-Cruz Biotechnology (mouse) and Cell Signaling Technology (rabbit).

### Inhibitors

Salubrinal (SAL), Cycloheximid (CHX), JNK3XIISR3576 (JNK3XII), and SB203580 (SB) were obtained from Calbiochem. Ly29400L (Ly) was from Cell Signaling. Bafilomycin, Cytochalasin D (Cyto D), and Cerulenin (Ceru) were from Sigma and Dynasore (Dyn) was from Tocris bioscience.

### Chemicals

RNase, propidium iodide, and puromycin were purchased from Sigma. NHS-fluorescein and EZ-Link Sulfo-NHS-LC-biotin were from Thermo Fisher Scientific and Streptavidin-Alexa 647 was obtained from Molecular Probes. Rapamycin was from Calbiochem.

### Toxins

α-toxin, internally radio-labeled α-toxin, streptolysin (SLO), and VCC were made as published elsewhere (10, 12).

### *S*. *aureus*

In this study, *S. aureus* strain DU1090 (55) and α-toxin producing *S. aureus* strain, plasmid transformed derivative of DU1090 (55), were employed and referred to as hla(−) and hla(+), respectively.

### Cells and culture

Mouse embryonal fibroblasts GCN2^−/−^ (56) were purchased from ATCC and PDV from CLS Cell Lines Service GmbH. MEFs eIF2α^S51A/S51A^ and corresponding control cells MEFs eIF2α^S51/S51^(57), MEFs Ppp1r15b^−/−^ (58), MEFs EeF2K^−/−^ (59), and MEFs ADAM10^−/−^ (60) were kindly provided by Heather Harding and David Ron, Randal Kaufman, Alexey Ryazanov, and Paul Saftig, respectively. All MEFs cell lines were cultured in DMEM GlutaMAX™-I medium with 10% fetal calf serum, 1% HEPES buffer, 1% penicillin/streptomycin, 1% MEM NEAA, and 55 mM 2-mercaptoethanol. Under these conditions, we did not note significant differences in morphology, viability, or growth rate. PDV (murine keratinocyte cell line) were grown in DMEM GlutaMAX™-I medium with 10% fetal calf serum, 1% HEPES buffer, 1% penicillin/streptomycin without MEM NEAA, and 2-mercaptoethanol. HaCaT (non-virally transformed HaCaT) (61) were cultured in DMEM/F-12 GlutaMAX™-I medium with 10% fetal calf serum, 1% HEPES buffer, and 1% penicillin/streptomycin. All media and medium additives were obtained from Gibco by life technologies™. BMDMs were isolated from C57BL/6J (B6/J) or B6.129S6-EIF2αk4^tm1.2Dron/j^ (GCN2^−/−^) mice (Charles River laboratories) and cultured in DMEM GlutaMAX™-I supplemented with 20% fetal calf serum and M-CSF by adding 10% supernatant of L929 cells.

### Western blot

For Western blots, cell lysates were mixed with 2× SDS-loading buffer [65 mM Tris, 10% (v/v) glycerol, 5% (v/v) 2-mercaptoethanol, 2% (w/v) SDS, and bromphenol blue] and boiled for 5 min at 95°C. Proteins were separated by SDS-PAGE (10%) and electroblotted onto nitrocellulose membrane. After blocking for 1 h at room temperature in BSA or skim milk in TBST [Tris 50 μM, NaCl 0.15M, Tween 0.1% (v/v)], membrane was incubated with a primal antibody in BSA or skim milk in TBST, washed three times in TBST and incubated with HRP-conjugated second antibody for 1 h at room temperature. After three washing steps, bound antibody was detected by ECL (Roche Applied Science).

### Puromycin incorporation

The assay has been described elsewhere (62). MEFs eIF2α^S51A/S51A^ cells were seeded at a density of 2 × 10^5^ cells/well into six-well plates. Cells were incubated as indicated with several concentrations of pVCC or α-toxin for 1 h at 37°C. Thereafter, 10 μg/ml puromycin was added and cells were incubated for an additional hour at 37°C. Subsequently, medium was removed; cells were washed with PBS and analyzed by Western blot using anti-Puromycin-antibody in 2.5% (w/v) skim milk in TBST.

### ATP-measurements

Measurements of intracellular ATP were performed, as described elsewhere (22).

### Potassium efflux measurements

For measurements of intracellular potassium, cells were seeded at a density of 3 × 10^5^ cells/well into six-well plates. Cells were incubated or not with 8 μM Rapamycin for 3 h before adding 10 μg/ml α-toxin. After 2 h incubation at 37°C, cells were washed three times on ice with cold Choline-chlorid-buffer and solubilized in 2 ml Choline-chlorid-buffer containing 0.5% Triton X-100 (63) for 30 min at room temperature under constant shaking. Potassium content of supernatants of centrifuged cell lysates was determined using Sherwood single channel flame photometer M401.

### Quantitation of surface-exposed or internalized α-toxin/ADAM10

Surface labeling, neutravidin pull-down, and fluorometric detection of α-toxin have been described previously (13). Variation of input (labeled S35-Met-α-toxin) was <1%; equal amounts of total protein were loaded.

### Quantification of internalized bacteria

The assay has been performed, as described elsewhere (64).

### Sub-G1 analysis

Cells were seeded at a density of 1 × 10^5^ cells/well into six-well plates and treated or not with 10 μg/ml α-toxin permanently for 48 h at 37°C. Cells were harvested including the detached cells in the supernatant, washed in PBS and centrifuged. Cells were resuspended and fixed in cold 70% ethanol in PBS/EDTA for 2 h at 4°C. Subsequently, the cells were washed and treated with RNase for 10 min at 37°C. Then, propidium iodide was added and cells were incubated for 5 min at room temperature. Eventually, cells were analyzed using a FACScan flow cytometer (BD Biosciences) and CellQuest software.

### Statistical analysis

Two-sided, unpaired Student’s *t*-test was employed to assess statistical significance of differences between mean values. Significance was assumed at *p* < 0.05.

## Conflict of Interest Statement

The authors declare that the research was conducted in the absence of any commercial or financial relationships that could be construed as a potential conflict of interest.
